# Injuries to pedal cyclists on New Zealand roads, 1988-2007

**DOI:** 10.1186/1471-2458-10-655

**Published:** 2010-10-30

**Authors:** Sandar Tin Tin, Alistair Woodward, Shanthi Ameratunga

**Affiliations:** 1 Section of Epidemiology and Biostatistics, School of Population Health, University of Auckland, Auckland, New Zealand; 2 School of Population Health, University of Auckland, Auckland, New Zealand

## Abstract

**Background:**

The risk of injury is one of the major barriers to engaging in cycling. We investigated exposure-based rates and profiles of traffic injuries sustained by pedal cyclists that resulted in death or hospital inpatient treatment in New Zealand, one of the most car dependent countries.

**Methods:**

Pedal cyclist traffic injuries were identified from the Mortality Collection and the National Minimum Dataset. Total time spent cycling was used as the measure of exposure and computed from National Household Travel Surveys. Analyses were undertaken for the periods 1988-91, 1996-99 and 2003-07 in relation to other major road users and by age, gender and body region affected. A modified Barell matrix was used to characterise the profiles of pedal cyclist injuries by body region affected and nature of injury.

**Results:**

Cyclists had the second highest rate of traffic injuries compared to other major road user categories and the rate increased from 1996-99 to 2003-07. During 2003-07, 31 injuries occurred per million hours spent cycling. Non-collision crashes (40%) and collisions with a car, pick-up truck or van (26%) accounted for two thirds of the cycling injuries. Children and adolescents aged under 15 years were at the highest risk, particularly of non-collision crashes. The rate of traumatic brain injuries fell from 1988-91 to 1996-99; however, injuries to other body parts increased steadily. Traumatic brain injuries were most common in collision cases whereas upper extremity fractures were most common in other crashes.

**Conclusions:**

The burden of fatal and hospitalised injuries among pedal cyclists is considerable and has been increasing over the last decade. This underscores the development of road safety and injury prevention programmes for cyclists alongside the cycling promotion strategies.

## Background

The popularity of cycling as a sport and recreation activity has increased in New Zealand over recent years [1,2]. In 2006, for the first time, more bicycles were imported into the country than cars [3,4]. However, a bicycle is rarely used as a mode of transportation and represents only 2% of total travel time [5]. The proportion of commuters using a bicycle has been declining since 1986 and less than 3% of the employed population cycled to work in 2006 [6]. The largest decline was observed among younger age groups and females [6,7].

One of the major barriers to engaging in cycling is the real and perceived risk of injury [8,9]. The most recent (2008) data from the Ministry of Transport, based on police reports, showed that ten cyclists were killed, 186 were seriously injured and many more suffered minor injuries due to police-reported crashes on public roads [10]. The estimated total social cost was approximately NZ$224 million [10]. However, these figures are unlikely to represent an accurate profile of cycling injuries because it is not clear how "serious injury" was defined, and crashes are generally reported to the police only if a motor vehicle is involved. Langley and colleagues found that only 22% of cyclists admitted to hospital following a crash on public roads appeared in the police crash data [11].

Earlier research using the mortality and hospitalisation data showed a decline in cycling injuries involving a collision with a motor vehicle from 1989 to 1998 [12]. This paper aimed (1) to assess exposure-based rates of on-road injuries to pedal cyclists that resulted in death or hospital inpatient treatment over the period 1988-2007, (2) to quantify differences in such rates in relation to other road users and by age, gender and body region affected, and (3) to describe cycling injury profiles using a modified Barell matrix [13].

## Methods

### Data sources

The data for this analysis were obtained from the National Minimum Dataset and the Mortality Collection maintained by the Ministry of Health's Information Directorate, and the Household Travel Survey Dataset maintained by the Ministry of Transport. Specific approval was not sought as the data were publicly available upon request and only anonymous data were used for all analyses.

#### National Minimum Dataset

This includes information about all day patients and inpatients discharged from all public hospitals and over 90% of private hospitals in New Zealand. The data collected include demographic information, diagnoses and diagnostic and therapeutic procedures. For all injury discharges, the circumstances of injury are coded according to the external causes of injury and poisoning codes (E codes) and the nature of injury is coded according to the International Classification of Diseases (ICD) [14]. ICD-9-CMA was used before July 1999 and ICD-10-AM afterward. It was reported that 5% of the principal diagnosis and 18% of the first four digits of the E-codes for hospital discharges during 1996-98 that were coded under ICD-9-CM and 14% of the principal diagnosis and 26% of the E-codes for hospital discharges during 2001-04 that were coded under ICD-10-AM were incorrect [15,16].

#### Mortality Collection

This includes information about all deaths registered in New Zealand from 1988 onwards. The data collected include demographic information and the underlying cause of death coded according to the ICD [14]. ICD-9-CMA was used before 2000 and ICD-10-AM afterward. Overseas research suggested that the inaccuracies of coding could be greater for death records compared to hospital discharge records, particularly among older people [17-19]; however, there is insufficient evidence in New Zealand to confirm this.

#### Household Travel Surveys

The three separate national surveys [20] collected information on daily personal travel, with the sampling frame comprising all residents (including children) in private dwellings in New Zealand. The entire survey methodology was piloted. The survey questions were developed for the New Zealand Ministry of Transport by AMPT Applied Research Pty Ltd (Sydney) and pre-tested on households from a range of socio-economic backgrounds.

The first survey was undertaken between 1 July 1989 and 30 June 1990 and included 8,719 people aged five and over. The second survey was carried out between 1 July 1997 and 30 June 1998 and included over 14,250 people of all ages. From 1 August 2003, an ongoing survey has been conducted each year, with the sampling frame comprising approximately 2,000 households (resulting in responses from about 3500 people) per year. Up to 30 June 2009, 25,471 people have participated in the third survey.

Full response rates (i.e., the percentage of eligible households in which all members participated fully in the survey) were 75%, 75% and 66% respectively for the first, second and third surveys and full and partial response rates (i.e., the percentage of eligible households in which one or more members participated fully in the survey) were 78%, 79% and 71% respectively.

In each survey, travel time was assessed by asking respondents to keep a record of the times and places of all their travel over a specified two-day period. Departure and arrival times of each trip leg were recorded, along with trip destination, travel mode and purpose. The use of a two-day travel period minimises respondent burden and reliance on memory, compared to using a week-long period. Shortly after the conclusion of the two-day period, an interviewer questioned each respondent about their travel using the travel record as a memory aid. Interviewers were trained to prompt the respondent to recall any trips (particularly short trips) which may not have been recorded on their memory jogger.

### Statistical Analysis

The rate of traffic injuries to pedal cyclists resulting in death or hospital inpatient treatment was calculated using the equation:

$$\text{Injury rate=}\frac{\text{Total number of cases of cycling injuries per year}}{\text{Total time spent cycling (million hours) per year}}$$

The annualised total time spent cycling was used as the measure of exposure and computed from the three travel surveys, covering the periods: 1 July 1989-30 June 1990, 1 July 1997-30 June 1998 and 1 August 2003-30 June 2008. The data were weighted to account for clustering by household and non-response to the survey.

Traffic injuries (i.e., injuries occurring on a public highway) among pedal cyclists were identified using the E-codes (ICD-9-CM: E810-819.65, E826.15, E826.95, E829-829.15; and ICD-10-AM: V10-18.3-9, V19.4-6, V19.9) [14]. The hospitalised sample was restricted to inpatient discharges from public hospitals as the majority of patients (over 97%) requiring acute inpatient treatment for injury are admitted to public hospitals [21-23]. In order to enhance the validity of the analyses, the inclusion criteria included: (a) patients with a principal diagnosis of injury only (ICD-10-AM: S00-T78), (b) patients admitted to hospital for one day or more and (c) first admissions only [22]. Cases aged under five years were excluded from the overall sample as they were not surveyed in the 1989/90 travel survey. The annualised numbers of cycling injuries were computed for the periods 1988-91, 1996-99 and 2003-07 to stabilise small cell sizes.

Traffic injuries to pedal cyclists were sub-classified according to the mechanism of injury into (a) a collision with a motor vehicle (ICD-9-CM: E810-819.6; ICD-10-AM: V12-V14.3-9, V19.4-6) and (b) others. For injuries that occurred between 2003 and 2007, more detailed mechanism as mentioned in the ICD-10-AM was reported.

Injury rates and 95% confidence intervals were presented in relation to other major road users (car/van drivers, ca/van passengers, motorcyclists and pedestrians) and by age, gender and body region affected. Based on the assumption that the number of injuries had a Poisson distribution, the confidence intervals were calculated by using the normal approximation (if number of injuries was more than 20 per year) or the exact probability function (if the number of injuries was 20 or less per year) [24].

A modified form of the Barell matrix was used to characterise the profiles of pedal cyclist injuries that occurred between 2003 and 2007 by body region affected and nature of injury. The Barell matrix comprises 36 body region rows and 12 nature of injury columns and places each injury ICD-9-CM code in a unique cell location [13]. For this analysis, the ICD-10-AM codes were mapped into the ICD-9-CM codes and classified into seven body regions - traumatic brain injury, other head, face and neck, spine and back, torso, upper extremity, lower extremity and others - and six injury natures - fracture, dislocation/sprains and strains, internal, open wound, contusion/superficial and others. The "multiple injury profiles" approach [25-27] was used and up to ten diagnoses per case were extracted from the dataset.

To ensure an adequate sample size, analyses were undertaken for all deaths and hospitalisations. A further analysis restricted the cases of interest to those with serious injuries defined as an injury resulting in a significant threat to life (i.e., consistent with an Abbreviated Injury Scale (AIS) [28,29] score of 3 or more) in order to minimise the effect of extraneous factors such as service utilisation in assessing trends [19]. The mapping to this AIS threshold was achieved using the Barell matrix categorisation [30]. SAS (release 9.1, SAS Institute Inc., Cary, NC) and Microsoft Office Excel 2003 were used for all analyses.

## Results

During 2003-07, 31 injuries occurred per million hours spent cycling; 20% had a serious injury suggesting a significant threat to life, consistent with an estimated AIS score of 3 or more (Table 1). Of the major road user categories, the injury rate for pedal cyclists was surpassed only by motorcyclists. The cycling injury rate fell from 1988-91 to 1996-99 and rose afterward; similar but less pronounced trends were observed when the sample was restricted to those with serious injuries and to those involved in a collision with a motor vehicle (Table 2). In contrast, a declining trend in injury rates was observed among other road users (Table 1).

**Table 1:** Annual numbers and rates of traffic injuries that resulted in death or hospital inpatient treatment

	Annual number of injuries			Annual number of injuries per million hours spent travelling (95% CI)		
	
Mode of travel	1988-91	1996-99	2003-07	1988-91	1996-99	2003-07
***Overall***						
Cyclists	941	512	682	25.61 (23.97, 27.25)	21.38 (19.53, 23.23)	30.74 (28.43, 33.04)
Car/van driver	2081	2051	1714	4.24 (4.06, 4.42)	3.22 (3.08, 3.36)	2.10 (2.00, 2.20)
Car/van passenger	1568	1428	1086	5.64 (5.36, 5.92)	4.67 (4.43, 4.91)	2.89 (2.71, 3.06)
Motorcyclist	1655	895	784	185.14 (176.22, 194.06)	161.77 (151.18, 172.37)	107.64 (100.11, 115.17)
Pedestrian	743	638	471	4.29 (3.98, 4.60)	3.40 (3.14, 3.67)	2.38 (2.17, 2.60)
***Serious injuries (AIS*≥*3*)**						
Cyclists	377	117	138	10.27 (9.23, 11.30)	4.86 (3.98, 5.75)	6.24 (5.21, 7.28)
Car/van driver	886	774	629	1.81 (1.69,1.93)	1.22 (1.13, 1.30)	0.77 (0.71, 0.83)
Car/van passenger	643	516	367	2.31 (2.13, 2.49)	1.69 (1.54, 1.83)	0.98 (0.88, 1.08)
Motorcyclist	483	273	190	54.05 (49.23, 58.87)	49.24 (43.39, 55.09)	26.11 (22.40, 29.82)
Pedestrian	362	254	187	2.09 (1.88, 2.31)	1.36 (1.19, 1.52)	0.95 (0.81, 1.08)

**Table 2:** Annual numbers and rates of collision vs. other traffic injuries to pedal cyclists that resulted in death or hospital inpatient treatment

	Annual number of injuries			Annual number of injuries per million hours spent travelling (95% CI)		
	
Mode of travel	1988-91	1996-99	2003-07	1988-91	1996-99	2003-07
***Total***						
Collision with a motor vehicle	349	185	189	9.51 (9.01, 10.01)	7.66 (7.11, 8.21)	8.47 (7.86, 9.07)
Others	592	327	493	16.11 (15.46, 16.76)	13.57 (12.84, 14.31)	22.02 (21.05, 22.99)
***Serious injuries (AIS*≥*3*)**						
Collision with a motor vehicle	166	55	61	4.51 (4.16, 4.85)	2.29 (1.99, 2.59)	2.74 (2.40, 3.09)
Others	211	62	78	5.75 (5.36, 6.13)	2.57 (2.25, 2.89)	3.49 (3.10, 3.87)

The most common mechanisms of cycling injuries that occurred between 2003 and 2007 were non-collision crashes (40.4% of all cases and 33.6% of cases with serious injuries) and collisions with a car, pick-up truck or van (25.5% of all cases and 38.5% of cases with serious injuries) (Table 3).

**Table 3:** Mechanism of traffic injuries to pedal cyclists that resulted in death or hospital inpatient treatment (2003-2007)

Mechanism	Overall Injuries (%)	Serious Injuries (%)
Collision with car, pick-up truck or van	25.51	38.49
Collision with heavy transport vehicle or bus	1.79	5.35
Collision with two- or three-wheeled motor vehicle	0.35	0.58
Collision with other non-motor vehicle	0.06	0.00
Collision with other pedal cycle	4.11	5.35
Collision with pedestrian or animal	1.00	1.30
Collision with fixed or stationary object	5.60	7.53
Noncollision transport crashes	40.35	33.57
Unspecified	21.23	8.10

**Figure 1:**
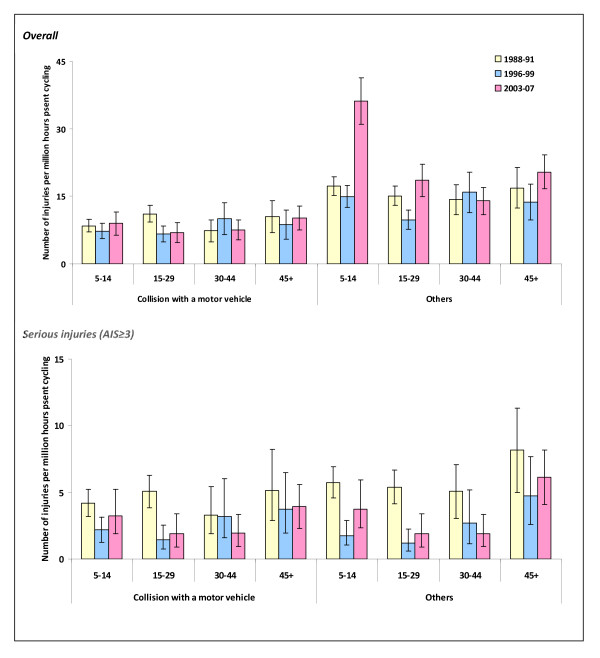
**Annual rates of traffic injuries to pedal cyclists that resulted in death or hospital inpatient treatment by age**.

**Figure 2:**
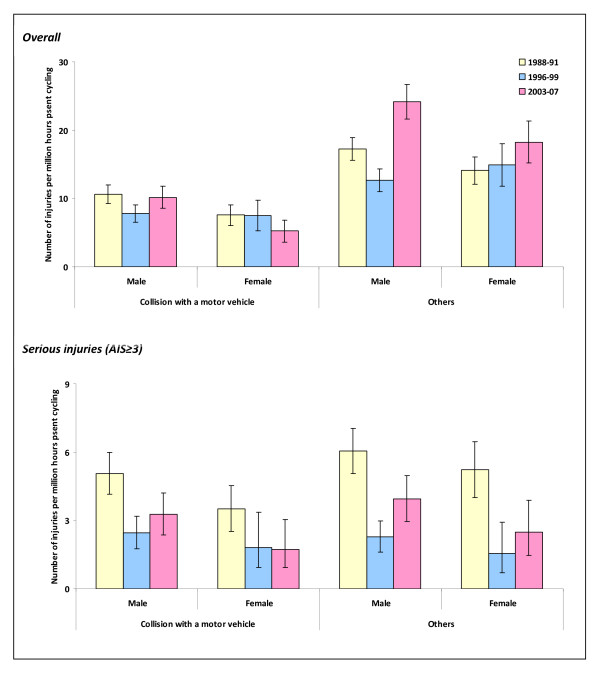
**Annual rates of traffic injuries to pedal cyclists that resulted in death or hospital inpatient treatment by gender**.

The highest rate of cycling injuries was observed among the 5-14 year olds (Figure 1). In this age group, from 1996-99 to 2003-07, there was a substantial increase in injury risk from crashes not involving a motor vehicle. However, this trend was not observed when analyses were restricted to those with serious injuries. Males had a higher rate of collision and other injuries compared to females (Figure 2).

**Figure 3:**
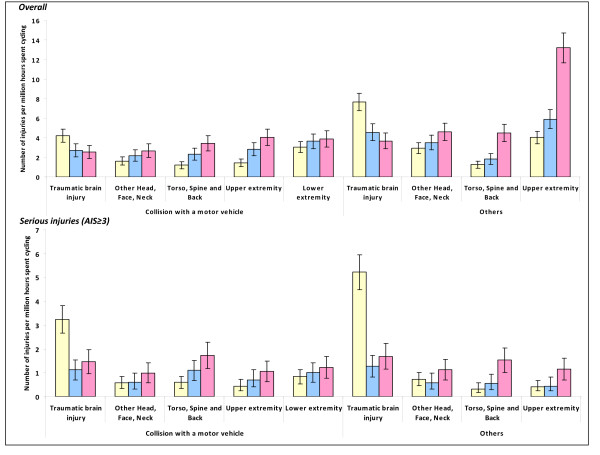
**Annual rates of traffic injuries to pedal cyclists that resulted in death or hospital inpatient treatment by body region affected**.

Compared to 1988-91, rates of traumatic brain injuries were lower in 1996-99 and 2003-07 (Figure 3). In contrast, there was an increasing trend over time in rates of injuries to other body parts. During 2003-07, the commonest injuries in crashes involving a collision with a motor vehicle were traumatic brain injuries (29.1%), open wounds in the head, face or neck (26.3%) and fractures in upper and lower extremities (25.9% and 24.7% respectively) (Table 4). In cycling injuries unrelated to motor vehicle collision, upper extremity fractures (40.3%) were most common, followed by open wounds in the head, face or neck and traumatic brain injuries (16.9% and 14.9% respectively). Traumatic brain injuries, lower extremity fractures and open wounds in the head, face or neck occurred in 28.2%, 19.2% and 18.7% respectively of cases with serious injuries (Table 5).

**Table 4:** Modified Barell body region by nature of injury diagnosis matrix for overall injuries (2003-07)

Body Region Injured	Fracture	Dislocation, Sprains & Strains	Internal	Open wound	Contusion/ superficial	Others
***Percent of cyclists injured in crashes involving a collision with a motor vehicle***						
Traumatic brain injury	6.48	0.00	29.05	0.00	0.00	0.00
Other Head, Face, Neck	3.66	0.21	0.00	26.33	11.91	0.63
Spine and back	12.64	2.93	1.25	0.00	0.00	0.00
Torso	11.81	1.46	11.81	2.30	8.36	1.67
Upper extremity	25.91	5.22	0.00	13.58	7.42	1.25
Lower extremity	24.66	5.22	0.00	13.17	14.73	2.09
Others	0.00	0.21	0.00	4.49	1.78	2.61

***Percent of cyclists injured in other crashes***						
Traumatic brain injury	2.50	0.00	14.90	0.00	0.00	0.00
Other Head, Face, Neck	3.05	0.16	0.00	16.88	9.31	0.16
Spine and back	3.33	1.35	0.59	0.00	0.00	0.00
Torso	4.28	0.91	4.91	1.15	3.65	1.39
Upper extremity	40.33	6.46	0.00	8.87	4.08	0.79
Lower extremity	11.17	6.46	0.00	8.84	10.58	2.14
Others	0.00	0.20	0.00	4.87	1.51	1.19

**Table 5:** Modified Barell body region by nature of injury diagnosis matrix for serious injuries (2003-07)

Body Region Injured	Fracture	Dislocation, Sprains & Strains	Internal	Open wound	Contusion/ superficial	Others
***Percent of cyclists with serious injuries (AIS ≥ 3)***						
Traumatic brain injury	11.25	0.00	28.16	0.00	0.00	0.00
Other Head, Face, Neck	5.20	0.19	0.00	18.68	7.81	0.37
Spine and back	8.36	2.42	2.51	0.00	0.00	0.00
Torso	11.71	3.07	14.78	0.65	3.07	0.46
Upper extremity	13.57	1.86	0.00	5.86	3.90	0.93
Lower extremity	19.24	1.86	0.00	5.76	7.62	1.58
Others	0.00	0.09	0.00	1.67	0.46	2.60

## Discussion

Our analysis showed that cyclists had the second highest rate of traffic injuries resulting in death or hospital inpatient treatment compared to other major road user categories when analysed in relation to time spent travelling. The cycling injury rate increased from 1996-99 to 2003-07. Non-collision crashes and collisions with a car, pick-up truck or van were responsible for about two-thirds of cycling injury deaths and hospitalisations during 2003-07. Children and adolescents aged under 15 years were at the highest risk of being involved in non-collision crashes although this pattern was not observed for serious injuries. The rate of traumatic brain injuries fell from 1988-91 to 1996-99; however, injuries to other body parts increased steadily. Traumatic brain injuries were most common in crashes involving a collision with a motor vehicle whereas upper extremity fractures were most common in other crashes.

The major strength of this study is the use of data from the three national datasets to quantify exposure-based rates of cycling injuries resulting in death or hospital inpatient treatment. However, some limitations should be kept in mind when interpreting the findings.

Injuries treated in emergency departments and private primary care facilities, or self-treated, were not included in this analysis. It has been proposed that such injuries be excluded in developing indicators of injury incidence due to incomplete ascertainment [19]. While such injuries may not pose a significant threat to life, it cannot be assumed that they will not pose a threat to longer-term disability. Ascertainment of relevant cases could also be affected by inaccuracies in diagnosis and external cause codes [15,16]. This could have a greater impact on time series analyses as the ICD9-CM was replaced with the ICD10-AM in 1999-2000. In addition, there were inconsistencies in the uploading of day cases to the National Minimum Dataset over time. To address this, we restricted the hospitalised sample to patients admitted for one day or more. It is also recognised that admission to hospital could be influenced by a number of factors including severity of injury, pre-existing co-morbidities, access to hospital services, professional practice and bed/theatre availability [19]. In this analysis, there is the potential for misclassification of injury severity due to the heterogeneity of the diagnostic categories included in the Barell matrix [31].

The estimates derived from the travel surveys may underestimate the total travel in New Zealand due to the exclusion of some people who travel a lot such as visitors, guests at hotels/motels, people who are not at home when surveyed and professional drivers (e.g., taxi and truck drivers). As relatively few bicycle trips were recorded compared to other modes, the cycling exposure information may be subject to a certain level of uncertainty. However, we did not anticipate any significant variation in accuracy of information collected over time as the same questions were used for all three surveys with high response rates although it is possible that the decrease in the response rate between the second and the third survey may impact on reliability of the data available.

Despite these limitations, the study presents some important findings to inform the development of road safety, injury prevention and trauma care programmes and services. Consistent with our findings, overseas research show that cyclists bear a higher risk than car drivers per time or distance travelled [32,33]. However, in a Dutch study, after adjusting for motorway journeys, nearly twice as many motorists are killed as cyclists per billion kilometres travelled and the probability of being admitted to hospital is similar for both modes of transport [34]. Although we were not able to control for biases such as differences in types of roads used by cyclists and drivers [33], our findings indicate that cyclists are likely to be at greater risk when travelling in the road environment of auto-centric countries like New Zealand.

The "safety in numbers" phenomenon suggests that the risk profile of cyclists may improve if more people cycle [35]. In New Zealand, the overall travel mode share for cycling declined steadily from 4% in 1989 to 1% in 2006 [36] while the annual distance driven in light 4-wheeled vehicles increased [37]. This may account for an increasing trend in the cycling injury rate in the last decade (in contrast to a steady decline in the injury rate of other road users) in our analysis. Despite this, cyclists have attracted relatively little attention in the road safety agenda. For example, the focus of the recently released "New Zealand's Road Safety Strategy" is on motorised transport [38].

We found that non-collision crashes accounted for two-fifths of the overall cycling injuries resulting in death or hospital inpatient treatment whereas collision with a motor vehicle accounted for almost two-fifths of serious cycling injuries. This extends previous research using emergency department data (reporting that 64-79% of crashes did not involve another vehicle [39-43]), hospital records (reporting that 30-66% of crashes involved a motor vehicle [44-46]) and mortality data (reporting that 72-97% of crashes involved a motor vehicle [47-51]). Efforts to prevent cycling injuries could be more successful if factors associated with collisions are identified and targeted (e.g., traffic calming, providing cycle ways, improving cyclist conspicuity, driver education and training) alongside factors that are likely to impact on cyclist only crashes (e.g., improving road surface, cyclist skills training).

Of particular concern are children and adolescents who have experienced the greatest increase in the risk of cycling injuries despite a substantial decline in the amount of cycling over the past two decades. The travel surveys show that from 1989/90 to 2005/08, the average time spent cycling per week decreased from 28 minutes to 8 minutes among those aged 5-12 years and from 52 minutes to 12 minutes among those aged 13-17 years [7]. Likewise, from 1989/90 to 2004/08, cycling to school declined from 12% to 4% while being driven to school increased from 31% to 55% [7]. Parents' safety concern is one of the main reasons why children don't cycle or walk to school [52-54] although a wide range of factors can influence such behaviour [55]. If more children are driven to school, they will have less opportunity to develop cycling and road safety skills and traffic will be increased, posing more danger and initiating a vicious circle that can have an adverse impact on risks of injury as well as levels of physical activity. To address this, some initiatives have been developed in New Zealand, including the Walking School Bus Programme [56,57], school-based cycle trains [58] and cyclist skills training [59].

Our analysis showed the declining trend in rates of traumatic brain injuries from 1988-91 to 1996-99. However, it is unclear whether this reflects the effectiveness of the mandatory all-age cycle helmet law implemented in January 1994 or simply reflects a general decline in all road injuries during that period. On the other hand, we found a steady increase in injuries to other body parts over the twenty year period. However, there is a relative dearth of research focusing on such injuries and potential protective measures such as extremity guards.

## Conclusions

The burden of injuries to pedal cyclists is considerable and has been increasing over the last decade. A range of comprehensive strategies that can enhance bicycle safety, are required alongside strategies that promote cycling given its recognised health and environmental benefits.

## Competing interests

There is no conflict of interest including financial competing interest.

## Authors' contributions

STT has contributed to acquisition, analysis and interpretation of data and drafting the manuscript. AW and SA have contributed to interpretation of data and revising the manuscript critically. All authors have given final approval of the version to be published.

## Authors' information

STT - MBBS, MPH, Research Fellow, Section of Epidemiology and Biostatistics, School of Population Health, University of Auckland, New Zealand

AW - MBBS, PhD, FAFPHM, Professor and Head of School, School of Population Health, University of Auckland, New Zealand

SA - MBChB, PhD, FAFPHM, Professor, Section of Epidemiology and Biostatistics, School of Population Health, University of Auckland, New Zealand

## Pre-publication history

The pre-publication history for this paper can be accessed here:

http://www.biomedcentral.com/1471-2458/10/655/prepub
